# Effect of AI-Based Natural Language Feedback on Engagement and Clinical Outcomes in Fully Self-Guided Internet-Based Cognitive Behavioral Therapy for Depression: 3-Arm Randomized Controlled Trial

**DOI:** 10.2196/76902

**Published:** 2026-01-05

**Authors:** Mirai So, Yoichi Sekizawa, Sora Hashimoto, Masami Kashimura, Hajime Yamakage, Norio Watanabe

**Affiliations:** 1 Department of Psychiatry Tokyo Dental College Tokyo Japan; 2 Research Institute of Economy, Trade and Industry Tokyo Japan; 3 United Health Communication Co., Ltd. Tokyo Japan; 4 Department of Psychology Faculty of Human Schiences Tokiwa University Ibaraki Japan; 5 Department of Medical Statistics Satista Co., Ltd. Kyoto Japan; 6 Department of Psychiatry Soseikai General Hospital Kyoto Japan

**Keywords:** Adherence, AI, AI-supported psychotherapy, artificial intelligence, CBT, depression, internet-based CBT, natural language processing, RCT, self-help intervention

## Abstract

**Background:**

Depression remains a major global cause of disability; yet, access to optimal mental health services is limited. Self-guided internet-based cognitive behavioral therapy (iCBT) offers a scalable alternative but is generally less effective than guided programs, showing limited antidepressant effects and incomplete symptomatic and functional recovery. Adherence remains a major barrier. Recent advances in artificial intelligence (AI), particularly natural language processing, enable automated advisory and empathic feedback that may enhance engagement and therapeutic impact. Although previous trials have reported promising effects, most used heterogeneous control conditions, making it difficult to isolate the specific contribution of AI within fully self-guided interventions.

**Objective:**

This randomized controlled trial evaluated whether natural language processing–based AI feedback integrated into a fully self-guided iCBT program improves clinical outcomes and engagement compared with an otherwise identical iCBT program without AI support.

**Methods:**

We recruited 1187 adults aged 20-60 years online and randomly assigned them to AI-augmented iCBT (AI-iCBT; n=396), iCBT without AI (n=397), or a waitlist control (n=394). Both active groups received 6 weekly sessions combining video-based psychoeducation and cognitive restructuring exercises. The AI-iCBT program additionally provided automated empathic and advisory feedback. The primary outcome was depressive symptom severity (Patient Health Questionnaire-9 [PHQ-9]) at week 7 and month 3, analyzed using mixed-effects models for repeated measures under an intention-to-treat framework. Secondary outcomes included a dichotomous PHQ-9 score of ≥10, Quick Inventory of Depressive Symptomatology, Generalized Anxiety Disorder-7, Sheehan Disability Scale, and weekly participation rates. Exploratory analyses assessed the impact of AI functions on engagement and antidepressant effects in the efficacy analysis set (EAS).

**Results:**

In intention-to-treat analyses, no significant between-group differences were observed in mean PHQ-9 scores at week 7 or month 3, whereas engagement analyses showed a significant group × week interaction, with AI-iCBT participants demonstrating consistently higher odds of weekly participation (odds ratio 1.23, 95% CI 1.09-1.39; *P*<.001). Exploratory analyses indicated that activation of the empathic feedback function strongly predicted adherence (odds ratio 9.99, 95% CI 5.80-17.21; *P*<.001), while advisory feedback was not significant. In EAS analyses, iCBT showed significant short-term improvement versus control at postintervention, whereas at follow-up, only AI-iCBT showed a significantly lower proportion of participants with a PHQ-9 score of ≥10 compared with control (difference –0.15, 95% CI –0.30 to –0.01; *P*=.046). No serious adverse events were reported.

**Conclusions:**

AI support significantly improved adherence to a fully self-administered program. In EAS analyses, AI-iCBT also showed a significantly lower proportion of participants with PHQ-9 score of ≥10 at follow-up compared with control. Empathic feedback emerged as a key mechanism for sustaining engagement, suggesting that AI communication may help maintain participation in scalable digital mental health interventions. Further research is required.

**Trial Registration:**

University Hospital Medical Information Network Clinical Trials Registry (UMIN-CTR) UMIN000019228; https://center6.umin.ac.jp/cgi-open-bin/ctr/ctr_view.cgi?recptno=R000022220

## Introduction

Depression is a leading global cause of disability [1], substantially impairing quality of life [2,3] and imposing a considerable economic burden, including medical expenses [4] and productivity losses [5]. The rising prevalence of depression, combined with a shortage of health care resources, places a significant strain on health systems and professionals in meeting the growing demand [6-9].

In response, technology-delivered self-help interventions have emerged as promising solutions for managing mental health difficulties. Most of these interventions are based on cognitive behavioral therapy (CBT) and are generally referred to as internet-based CBT (iCBT) [10,11]. iCBT can be delivered either with therapist support (guided) or without therapist support (unguided, self-directed). Compared to traditional face-to-face therapy, iCBT offers major advantages in terms of accessibility, availability, and cost-effectiveness for both patients and providers. Furthermore, its online format provides benefits related to privacy, confidentiality, and anonymity, which can help reduce the stigma often associated with seeking mental health care [12,13].

While iCBT produces clinically meaningful symptom improvements, remission rates tend to be modest (approximately 30%-35%). An individual participant data meta-analysis reported a remission rate of 35.2% and a response rate of 56% [14]. Large-scale individual participant data network meta-analyses have consistently shown that guided iCBT yields higher response and remission rates than unguided formats, reflecting the challenges of engagement and dropout in fully self-administered programs [15-19].

This study specifically examines the unguided, fully self-administered format. Such interventions enable users to manage their symptoms independently and offer potential benefits such as reducing costs, alleviating the burden on health care providers, and improving access to mental health services in regions where such services are difficult to obtain [9,16]. Previous research has demonstrated that sociodemographic factors, such as age and sex, are associated with dropout risk in iCBT [20-22].

Nevertheless, if the effectiveness and engagement of unguided iCBT could be enhanced, the benefits of structured self-help materials would not be limited to fully self-administered interventions but would also extend to guided and blended formats. When patients acquire skills and knowledge through self-help modules, they can participate more effectively in therapist-led sessions, thereby enhancing overall treatment outcomes [23]. Strengthening such “self-help effects” not only amplifies therapeutic gains in guided and blended care but also reduces the time and workload required from therapists. By improving scalability and cost-effectiveness, it further increases the feasibility of implementation in routine practice [24,25]

To address these challenges, natural language processing (NLP), an artificial intelligence (AI) technology that enables the understanding and generation of human language, has increasingly been applied to enhance adherence and engagement through tailored feedback [26-28]. Whereas conventional unguided iCBT typically provides static or generic responses, in this study, we used an NLP-enabled iCBT program with automated advisory and empathic functions. This allows the system to generate advisory and empathic feedback in response to user input, potentially addressing both emotional and procedural barriers simultaneously.

Despite its promise, the specific therapeutic contributions of NLP remain unclear. Previous studies have frequently used heterogeneous control conditions such as waitlists, no intervention, treatment as usual, bibliotherapy, or conversational computer programs [28-32]. This heterogeneity makes it difficult to determine whether NLP provides distinct therapeutic benefits or merely functions as an active placebo by enhancing user expectations.

Against this background, the aim of this study was to conduct a randomized, parallel-group exploratory trial directly comparing 2 unguided, fully self-administered iCBT programs that were identical except for the presence or absence of NLP feedback. This design allowed us to evaluate the therapeutic contribution of NLP within a self-help framework in a blinded comparison of the 2 intervention groups.

## Methods

### Overview

This study was a 3-arm randomized controlled trial, with double-blinding between the AI-augmented iCBT (AI-iCBT) and iCBT groups, while the waitlist control group was unblinded. The intervention arms consisted of AI-iCBT, an unguided, fully self-administered iCBT program incorporating NLP feedback, and iCBT, an unguided, fully self-administered program without NLP feedback. These 2 arms are hereafter collectively referred to as “unguided iCBT.” The waitlist group served as the control condition.

### Study Participants

Invitation emails were sent to all monitors registered with Nikkei Research Inc, with the aim of recruiting at least 900 participants. Interested individuals were directed to complete an online screening survey, which included the Patient Health Questionnaire-9 (PHQ-9) [33,34], to determine eligibility.

### Eligibility Criteria

Inclusion and exclusion criteria are provided in Textbox 1.

**Eligibility criteria.**

**Inclusion criteria**

- Aged 20-60 years (to target the working-age population and exclude older adults with lower digital literacy)
- Had access to the internet
- Baseline Patient Health Questionnaire-9 score of ≥5 (this threshold was selected to avoid floor effects and ensure adequate symptom levels for change detection) [33,35,36]
- Ability to understand Japanese

**Exclusion criteria**

- Presence of medical conditions precluding participation as determined by a physician
- Concurrent participation in another cognitive behavioral therapy program
- Diagnosis of schizophrenia
- Severe suicidal ideation
- Diagnosis of dementia
- Substance dependence in the past 12 months (excluding smoking)

Eligible participants were randomly assigned to 1 of the 3 groups. Immediately before the intervention, PHQ-9 scores were reassessed; individuals with scores <5 were excluded from the efficacy analysis set (EAS) but remained in the overall study population. The detailed definition of the EAS is provided in the Analytic Strategy section.

### Intervention

**Figure 1:**
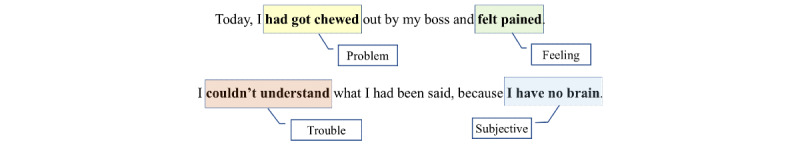
Examples of expressions extracted from the natural language processing corpus and categorized into 4 domains: Problem, Trouble, Feeling, and Subjective.

**Figure 2:**
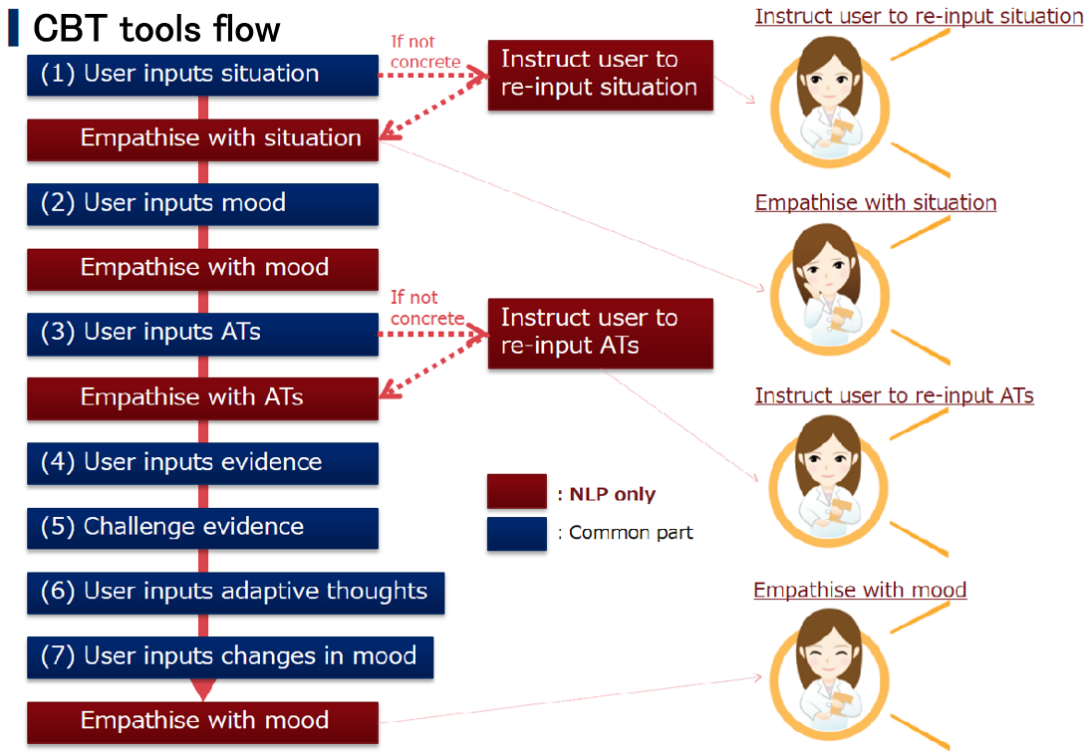
Workflow of artificial intelligence-guided internet-based cognitive behavioral therapy (CBT), showing the structured 7-step cognitive restructuring exercise with automated prompts and feedback. AT: automatic thought; NLP: natural language processing.

The AI-augmented iCBT program, developed by NEC Solution Innovators, Ltd (Tokyo), integrates an NLP module trained on 28,718 prior iCBT records from Japanese users. The entire program was delivered in Japanese, and Figure 1 presents an English-translated version of the original Japanese interface for publication purposes. The program includes a self-guided cognitive restructuring (CR) exercise, where users complete a 7-column thought record to address cognitive distortions. The NLP system processes user inputs, including situation, automatic thoughts, and feelings, referencing a corpus of past responses (Figure 1). It provides 2 types of automated feedback with text and phonation: (1) empathetic messages delivered through an animated character whose expressions, such as smiling or showing sadness, are synchronized with the message content, and (2) advisory messages offering guidance to refine inputs or direct users to appropriate exercises, including suggestions to revise content if the user’s input was unclear or misplaced (eg, a feeling given instead of a thought; Figure 2).

**Figure 3:**
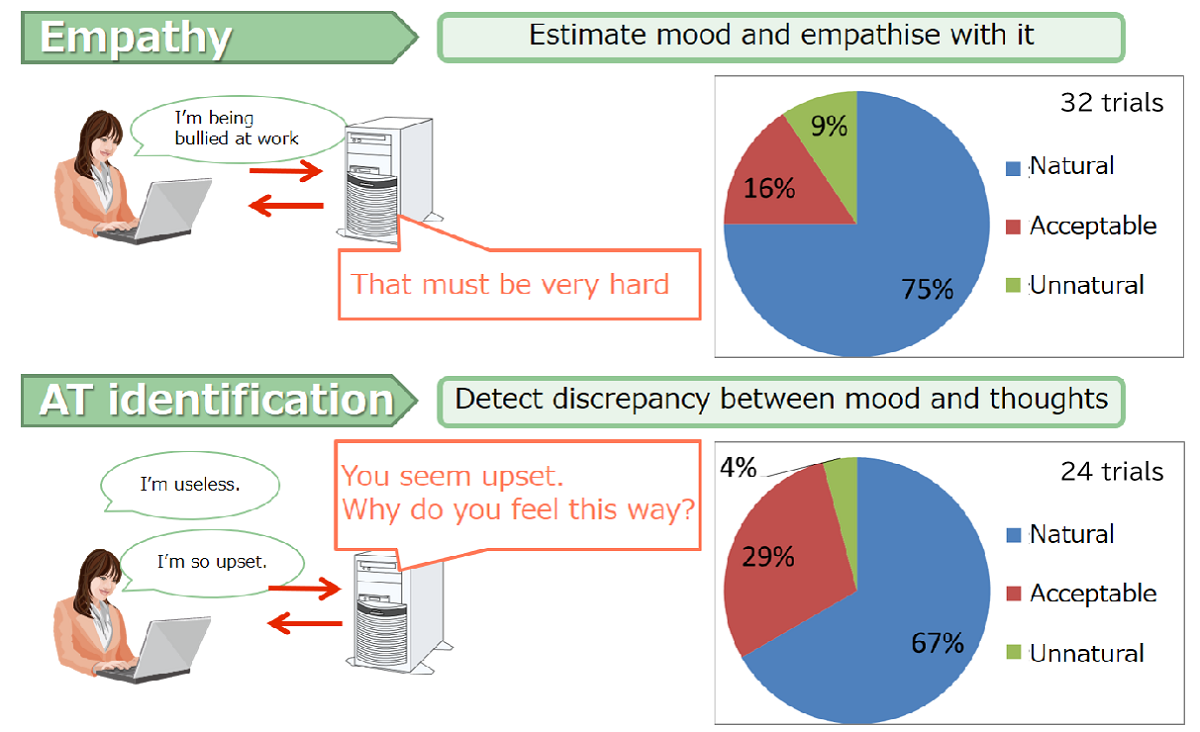
User acceptability ratings of natural language processing–generated feedback for empathy and automatic thought identification. AT: automatic thought.

In contrast, the non-AI iCBT program retained the same structure but provided only neutral, noncontextual responses, such as generic phrases like “Uh-huh” with neutral facial expressions. Both AI-iCBT and iCBT programs were otherwise identical in content, using the same validated 6-session self-guided iCBT package. This iCBT program has previously demonstrated significant antidepressant effects in a randomized controlled trial among working adults (n=60 per group), compared with a waitlist control, with a medium to large effect size (Cohen *d*=0.65; *P*<.005) [37]. This package consisted of 6 weekly sessions, each including a 15-minute video-based psychoeducation module covering standard CBT principles such as behavioral activation and problem-solving, along with a weekly CR exercise in which users applied learned techniques. In this trial, the only difference was the addition of the NLP feedback system. The program was available on both smartphones and PCs. The AI-enhanced features, which were designed in advance to improve user engagement and response accuracy, exhibited high usability, with low dissatisfaction rates reported for both the empathetic (3/32, 9.4%) and advisory (1/24, 4.2%) feedback functions (Figure 3).

### Randomization and Masking

The registered participants were randomly and concurrently assigned to either the AI-iCBT, iCBT, or waitlist groups using a computer-generated random sequence provided by an independent third party. Stratified randomization was applied based on age (≤40 vs >40 years), sex, and baseline PHQ-9 score (≤9 vs >9), as baseline symptom severity has been shown to influence treatment outcomes in self-guided iCBT [23]. Participants in the waitlist group were aware of their allocation and were therefore unblinded, whereas those in the AI-iCBT and iCBT groups were told only that they would participate in iCBT using “the latest technology,” without disclosure of their specific group assignment. Accordingly, blinding was implemented between the 2 intervention groups.

### Study Procedures

Automated email reminders were sent to participants twice weekly during the 7-week intervention period. Each week, participants in the intervention groups were required to (1) view an online psychoeducational CBT module and (2) perform their allocated (AI-iCBT or iCBT) CR exercise at least once (6 times or more in total). Waitlist participants did not undergo any exercises during this period. All participants were required to complete assessments at baseline, postintervention (week 7), and follow-up (month 3 after postintervention). All intervention and assessment procedures, including attendance and outcome measures, were conducted online.

### Outcomes

All primary and secondary outcomes were analyzed based on the intention-to-treat (ITT) population, which included all randomized participants.

#### Primary Outcome

The primary outcome was the mean PHQ-9 score, assessed at baseline, week 7 (postintervention), and month 3 (follow-up). The PHQ-9 is a widely used self-report measure of depressive symptoms (range 0-27, higher scores indicating greater severity), originally developed by Kroenke et al [33] and validated in Japanese [38].

#### Secondary Outcomes

Secondary outcomes include (1) proportion of participants with PHQ-9 scores ≥10 (a conventional cutoff for probable major depression) [33,39]. Although not selected as the primary outcome in this study, such binary outcomes are often considered clinically meaningful, as they reflect remission from a diagnostic threshold [40-43]. (2) Quick Inventory of Depressive Symptomatology-Japanese version (QIDS-J) [43,44]—a self-report scale of depressive symptom severity. (3) Generalized Anxiety Disorder-7 (GAD-7) [45,46]—a self-report questionnaire measuring generalized anxiety symptoms. (4) Sheehan Disability Scale (SDS) [47,48], which evaluates functional impairment in work, social, and family life, with SDS ≥10 commonly used as a pragmatic threshold in clinical trials [49].

Engagement outcome included weekly CR exercise attendance rate (defined as attending at least one session per week) in the 2 intervention groups. Program satisfaction at week 7 was assessed with the Client Satisfaction Questionnaire-8 (CSQ-8) [50,51], for which the Japanese version has demonstrated reliability and validity.

All outcomes except engagement and satisfaction were assessed at baseline, week 7, and month 3.

### Analytic Strategy

#### Overview

In this study, all primary and secondary analyses were conducted in the ITT population, defined as all randomized participants. In addition, 2 exploratory analyses were performed: (1) as an ad hoc exploratory analysis, we examined which AI feedback function (empathy or advisory) contributed more to enhancing engagement, and (2) as an additional exploratory analysis, we assessed continuous and binary PHQ-9 outcomes within the EAS.

#### Engagement-Enhancing Factors

For this analysis, the 2 intervention groups were combined, and the presence or absence of empathy and advisory feedback during week 1 was examined in relation to engagement from weeks 2 to 6, defined as completing at least 1 exercise per week. The detailed statistical methods are described in the Statistical Analysis section.

#### Efficacy Analysis Set

The EAS was defined as participants with a baseline PHQ-9 score of ≥5 and completion of at least 3 out of the 6 weekly sessions. Participants with a baseline PHQ-9 score of <5 (minimal symptoms) were excluded, as their inclusion could reduce the power to detect change and dilute the mean effects [52,53]. Furthermore, previous research has demonstrated a dose-response relationship in iCBT, with clinical benefits emerging after completing approximately half of the modules; therefore, the minimum attendance criterion was set at 3 of 6 sessions [54,55].

### Statistical Analysis

#### Overview

The sample size was estimated based on an assumed effect size of 0.10 (Cohen *d*) between the AI-iCBT and iCBT groups, given the absence of directly comparable prior studies. A dropout rate of 50% was anticipated based on patterns observed in similar previous studies. The power was set at 80% with a 2-sided significance level of α=.05. As this was an exploratory study, no adjustment for multiplicity was applied, and nominal *P* values were reported.

The primary and secondary analyses were conducted according to the ITT principle, including all randomized participants. Baseline demographic and clinical characteristics were compared across groups using 1-way ANOVA or chi-square tests.

Continuous outcomes (PHQ-9, QIDS-J, GAD-7, and SDS) were analyzed using a mixed-effects model for repeated measures (MMRM), with intervention, time, and intervention × time interaction as fixed effects, assuming an unstructured covariance structure. Results are presented as least squares means with 95% CIs.

Binary outcomes (PHQ-9 ≥10) were analyzed using generalized linear mixed models (GLMMs) with a binomial distribution and logit link, including intervention, time, and their interaction as fixed effects, and subject as a random effect. Estimated proportions and their 95% CIs were reported. Missing data for the outcomes were handled under the missing at random assumption within the MMRM and GLMMs framework.

CR exercise participation rates (defined as at least 1 completion per week) in the intervention groups were analyzed using GLMMs with a logit link, including intervention, week (as a continuous variable), and intervention × week interaction as fixed effects.

#### Exploratory Analyses

The following two exploratory analyses were conducted.

##### Engagement-Enhancing Factors

The dependent variable was defined as achieving at least 1 CR exercise per week across all weeks from week 2 to week 6 (yes/no). Independent variables were the presence or absence of empathy or advisory feedback during week 1. Covariates included age, sex, marital status, education, employment status, history of psychiatric and physical treatment, baseline PHQ-9 score, and intervention group (AI-iCBT vs iCBT), as group differences could confound the association of interest. Analyses were performed using generalized estimating equations logistic regression models to account for within-subject correlation and to estimate population-averaged effects.

##### Efficacy Analysis Set

In the EAS (participants with a baseline PHQ-9 score of ≥5 and completion of ≥3 sessions), continuous and binary PHQ-9 outcomes were additionally adjusted for age, sex, baseline PHQ-9 score, and medical history as covariates to account for potential group imbalances in the restricted sample.

#### Sensitivity Analyses

##### Associate Factors of Low Adherence

To explore potential factors associated with dropout, we compared baseline characteristics between EAS participants who attended ≥3 sessions and those who attended <3 sessions, given the high attrition typically observed in self-guided digital interventions.

##### Alternative Definition of Caseness

We conducted an exploratory analysis on the binary PHQ-9 outcome in the EAS, applying a stricter definition of depression severity: a PHQ-9 score of ≥10 plus at least 1 core symptom (depressed mood or anhedonia) [56,57], together with an SDS score of ≥10 as an indicator of functional impairment [49,58-60].

All analyses were performed using IBM SPSS Statistics version 26.0.

### Reporting Standards

Reporting of this trial followed the CONSORT (Consolidated Standards of Reporting Trials) 2010 statement [61] and the CONSORT-EHEALTH (Consolidated Standards of Reporting Trials of Electronic and Mobile Health Applications and Online Telehealth) checklist [62] for internet-based interventions. The completed CONSORT-EHEALTH checklist is submitted as Multimedia Appendix 1.

### Ethical Considerations

This study was reviewed and approved by the Hiramatsu Memorial Hospital Ethics Committee (approval number 20150807). All participants provided informed consent electronically prior to enrollment after reading an online information sheet describing the study purpose, procedures, potential risks, and voluntary nature of participation. Participants were informed that they could withdraw at any time without penalty.

The trial was prospectively registered in the University Hospital Medical Information Network Clinical Trials Registry.

All data were anonymized prior to analysis to ensure confidentiality. No personally identifiable information was accessible to the research team. Participants who completed the final assessment received a ¥500 (US $4.5) gift voucher as compensation. No identifiable images or other personal data are presented in this manuscript.

## Results

### Study Participants

**Figure 4:**
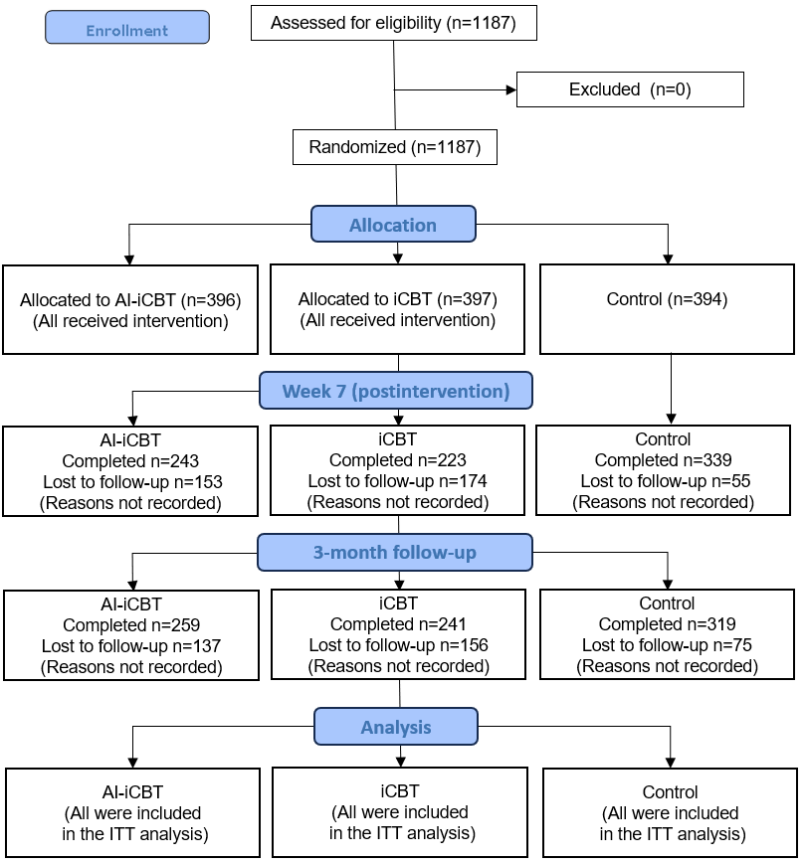
CONSORT (Consolidated Standards of Reporting Trials) 2010 flow diagram of participant enrollment, allocation, follow-up, and analysis. AI-iCBT: artificial intelligence–augmented internet-based cognitive behavioral therapy; iCBT: internet-based cognitive behavioral therapy; ITT: intention-to-treat.

A total of 1187 participants were eligible and randomly allocated to the AI-iCBT (n=396), iCBT (n=397), or waitlist (n=394) groups (ITT population; see Figure 4 for the CONSORT flow diagram). Baseline demographic and clinical characteristics are summarized in Table 1. The mean age was 43.50 (SD 9.85) years, and 699 (58.8%) of 1187 participants were male. Across the 3 groups, demographic and clinical characteristics were well balanced, with no significant differences in depressive symptom severity (PHQ-9: *P*=.56; QIDS-J: *P*=.74). No significant baseline differences were found between the AI-iCBT and iCBT groups, confirming the comparability of the 2 active interventions.

Figure 4 shows the flow of participants through the trial, including the numbers assessed for eligibility, randomized, allocated to each study arm (AI-iCBT, iCBT, control), completing follow-up assessments at week 7 and month 3, and included in the ITT analysis.

**Table 1:** Participants’ characteristics.

Characteristic		Total (N=1187)	AI-iCBT^a^ (n=396)	iCBT^b^ (n=397)	Control (n=394)	*P* value (overall)		*P* value (AI-iCBT vs iCBT)^c^	
**Sex, n (%)**							.96^d^		N/A^e^
	Male	698 (58.8)	232 (58.6)	232 (58.4)	234 (59.4)				
	Female	489 (41.2)	164 (41.4)	165 (41.6)	160 (40.6)				
**Age (years)**							.81^f^		N/A
	Mean (SD)	43.5 (9.9)	43.6 (9.5)	43.2 (9.9)	43.6 (10.1)				
	Median (IQR)	44 (36-52)	44 (36.8-51)	44 (36-52)	45 (36-52)				
	Minimum-Maximum	20-60	20-60	20-60	20-60				
**Marital status, n (%)**							.28^d^		N/A
	Married	665 (56)	226 (57.1)	219 (55.2)	220 (55.8)				
	Divorced	76 (6.4)	29 (7.3)	17 (4.3)	30 (7.6)				
	Bereaved	7 (0.6)	2 (0.5)	4 (1)	1 (0.3)				
	Single	439 (37)	139 (35.1)	157 (39.5)	143 (36.3)				
**Educational background, n (%)**							.63^d^		N/A
	Junior high school	7 (0.6)	3 (0.8)	1 (0.3)	3 (0.8)				
	High school	239 (20.1)	87 (22)	84 (21.2)	68 (17.3)				
	Junior college or technical	216 (18.2)	71 (17.9)	73 (18.4)	72 (18.3)				
	University or postgraduate	725 (61.1)	235 (59.3)	239 (60.2)	251 (63.7)				
**Employment status, n (%)**							.78^d^		N/A
	Working	972 (81.9)	320 (80.8)	324 (81.6)	328 (83.2)				
	Unemployed (seeking)	79 (6.7)	27 (6.8)	30 (7.6)	22 (5.6)				
	Unemployed (not seeking)	136 (11.5)	49 (12.4)	43 (10.8)	44 (11.2)				
**Medical history, n (%)**							.13^d^		N/A
	No relevant history	918 (77.3)	316 (79.8)	296 (74.6)	306 (77.7)				
	Ambulatory	258 (21.7)	74 (18.7)	97 (24.4)	87 (22.1)				
	Hospitalized	11 (0.9)	6 (1.5)	4 (1)	1 (0.3)				
**Mental history, n (%)**							.93^d^		N/A
	In treatment	129 (10.9)	46 (11.6)	44 (11.1)	39 (9.9)				
	Treated	184 (15.5)	61 (15.4)	59 (14.9)	64 (16.2)				
	No relevant history	874 (73.6)	289 (73)	294 (74.1)	291 (73.9)				
	PHQ-9^g^ score ≥10	428 (36.1)	142 (35.9)	143 (36)	143 (36.3)	.99^d^		N/A	
**Baseline scale score, mean (SD)**									
	PHQ-9	8.7 (5.2)	8.8 (5.2)	8.5 (5.2)	8.9 (5.1)	.56^f^		.41^f^	
	QIDS-J^h^	8.7 (4.9)	8.8 (4.9)	8.6 (4.9)	8.8 (4.7)	.74^f^		.49^f^	
	GAD-7^i^	6.0 (4.6)	6.0 (4.4)	5.9 (4.7)	6.2 (4.7)	.59^f^		.93^f^	

^a^AI-iCBT: artificial intelligence–augmented internet-based cognitive behavioral therapy.

^b^iCBT: internet-based cognitive behavioral therapy.

^c^*P* values represent pairwise comparisons between AI-iCBT and iCBT groups.

^d^*P* value is based on the chi-square test.

^e^N/A: not applicable.

^f^*P* value is based on ANOVA.

^g^PHQ-9: Patient Health Questionnaire-9.

^h^QIDS-J: Quick Inventory of Depressive Symptomatology-Japanese version.

^i^GAD-7: Generalized Anxiety Disorder-7.

### Primary and Secondary Outcomes (ITT Population)

The primary outcome, the mean score on the PHQ-9, did not show statistically significant between-group differences compared with the control group at either week 7 or month 3 (AI-iCBT vs control: least squares mean difference –0.47, 95% CI –1.13 to 0.18; *P*=.16; Cohen *d*=–0.10; iCBT vs control: least squares mean difference –0.62, 95% CI –1.28 to 0.04; *P*=.07; Cohen *d*=–0.13; Table 2). No significant differences were observed between the AI-iCBT and iCBT groups. Nevertheless, both intervention groups showed significant within-group reductions from baseline at week 7 and month 3 (all *P*<.001), indicating that depressive symptoms improved over time in both groups.

**Table 2:** Primary outcome: mean Patient Health Questionnaire-9 scores at baseline, week 7, and month 3 (intention-to-treat population). Values are least squares (LS) means with 95% CIs estimated using a mixed model for repeated measures. Between-group comparisons are shown.

Time point	AI-iCBT^a^ group, LS mean (95% CI)	iCBT^b^ group, LS mean (95% CI)	Control group, LS mean (95% CI)	AI-iCBT vs control, Δ (95% CI)	iCBT vs control, Δ (95% CI)
Baseline	8.79 (8.47 to 9.11)	8.73 (8.41 to 9.05)	8.81 (8.48 to 9.13)	—^c^	—
Week 7	7.90 (7.49 to 8.31)	7.75 (7.33 to 8.18)	8.28 (7.94 to 8.63)	–0.37 (–1.02 to 0.29); *P*=.27	–0.46 (–1.13 to 0.21); *P*=.18
Month 3	7.37 (6.98 to 7.77)	7.17 (6.76 to 7.58)	7.86 (7.50 to 8.22)	–0.47 (–1.13 to 0.18), *P*=.16	–0.62 (–1.28– to 0.04); *P*=.07

^a^AI-iCBT: artificial intelligence–augmented internet-based cognitive behavioral therapy.

^b^iCBT: internet-based cognitive behavioral therapy.

^c^Not available.

**Figure 5:**
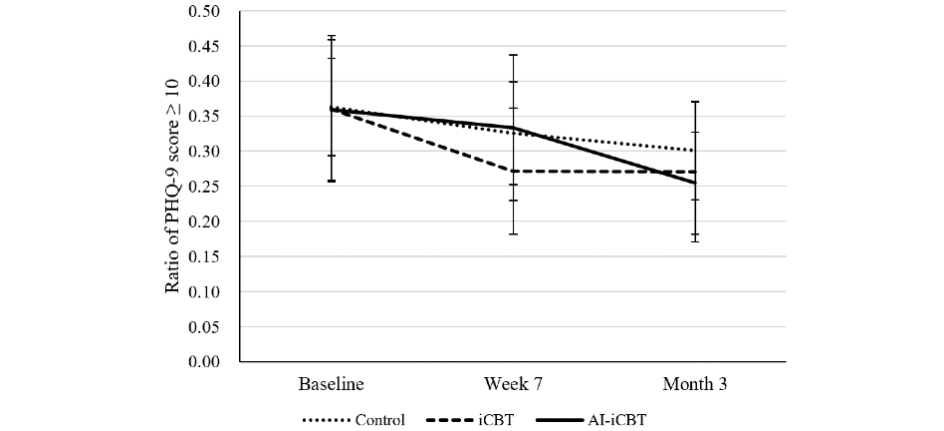
Secondary outcome: proportion of participants with Patient Health Questionnaire-9 (PHQ-9) scores ≥10 at baseline, week 7, and month 3 (intention-to-treat population). Estimated proportions and 95% CIs were derived from generalized linear mixed models with a logit link, including effects for group, time, and their interaction. AI-iCBT: artificial intelligence–augmented internet-based cognitive behavioral therapy; iCBT: internet-based cognitive behavioral therapy.

For the secondary binary outcome of PHQ-9 ≥10, the overall proportion decreased over time across all groups (Figure 5). At month 3, the proportion was numerically lower in the AI-iCBT group compared with the control group, but between-group differences were not statistically significant in the ITT analysis (Multimedia Appendix 2).

Similar patterns were observed for other secondary measures. QIDS-J and GAD-7 scores improved significantly within both intervention groups but without significant between-group differences. SDS scores showed modest reductions but did not significantly differ from control. Full secondary outcome results are provided in Multimedia Appendix 3.

### Engagement and User Satisfaction

#### Overview

**Figure 6:**
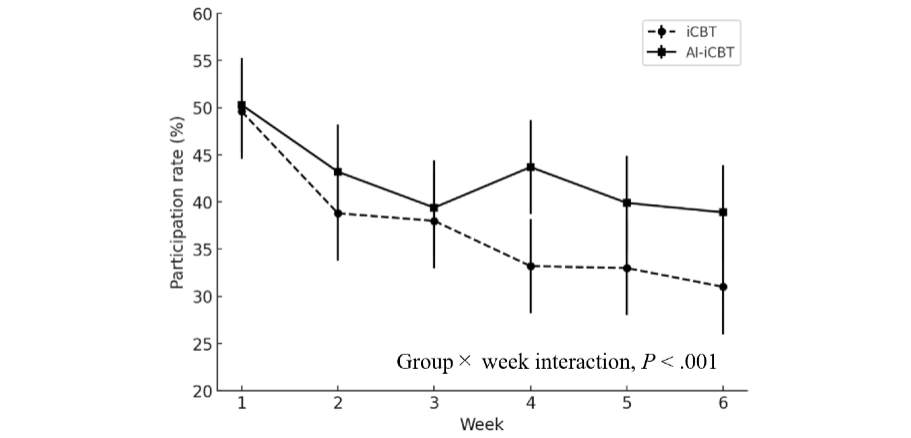
Engagement outcome: weekly participation rates in the artificial intelligence–augmented internet-based cognitive behavioral therapy (AI-iCBT) and internet-based cognitive behavioral therapy (iCBT) groups during weeks 1-6 (intention-to-treat population). Participation was defined as completion of at least 1 cognitive restructuring exercise per week. Error bars indicate 95% CIs.

As illustrated in Figure 6, the CR exercise participation rate decreased significantly over time across both intervention groups (odds ratio [OR] 0.751, 95% CI 0.692-0.815; *P*<.001). Participation began at only about half of participants in week 1 and declined further, dropping more steeply in the iCBT group, which fell to around 30% by week 6. In contrast, the AI-iCBT group retained somewhat higher engagement, remaining closer to the low 40% range by week 6, suggesting that AI support helped sustain participation over time. Between-group differences over time were examined using GLMM with a logit link, which showed a significant group × week interaction favoring AI-iCBT (OR 1.23, 95% CI 1.09-1.39; *P*<.001; Table 3). Analyses included all randomized participants in the intervention groups.

**Table 3:** Engagement outcome: mixed-effects logistic regression results for weekly cognitive restructuring exercise participation rates (intention-to-treat population). The generalized linear mixed model with a logit link included fixed effects for group, week (continuous and centered), and their interaction (group × week).

Effect	Reference	Odds ratio (95% CI)	*P* value
Group (AI-iCBT^a^ vs iCBT^b^)	iCBT	0.807 (0.370-1.758)	.59
Week (continuous, centered)	—^c^	0.751 (0.692-0.815)	<.001
Group × week	—	1.229 (1.090-1.386)	<.001

^a^AI-iCBT: artificial intelligence–augmented internet-based cognitive behavioral therapy.

^b^iCBT: internet-based cognitive behavioral therapy.

^c^Not available.

#### User Satisfaction

Assessed at week 7 with the CSQ-8, averaged about 21 out of 32 points in both intervention groups, indicating a moderate to good level of satisfaction. No significant difference was observed between AI-iCBT and iCBT (Multimedia Appendix 4).

#### Exploratory Analysis of Engagement-Enhancing Factors

Activation of the empathy function was significantly associated with higher participation (OR 9.99, 95% CI 5.80-17.21; *P*<.001). In contrast, activation of the advisory function was not significantly associated with engagement (OR 2.37, 95% CI 0.96-5.83; *P*=.06). Detailed adjusted results are provided in Multimedia Appendix 5.

#### Exploratory EAS Analysis

In the exploratory EAS analysis, based on the ITT population, 312/396 (78.8%) in the AI-iCBT group, 312/397 (78.6%) in the iCBT group, and 317/394 (80.5%) in the control group had a baseline PHQ-9 score of ≥5. Among the ITT population, the proportion of participants who attended 3 or more sessions was 188/396 (47.5%) in the AI-iCBT group and 158/397 (39.8%) in the iCBT group. Furthermore, the proportion of participants with a baseline PHQ-9 score of ≥5 who attended 3 or more sessions (EAS) was 149/396 (37.6%) in the AI-iCBT group, 134/397 (33.8%) in the iCBT group, and 317/394 (80.5%) in the control group.

**Figure 7:**
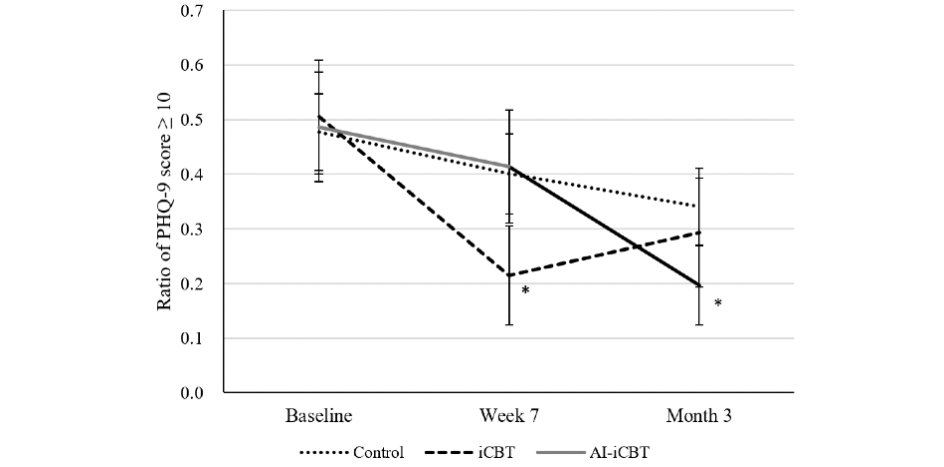
Exploratory outcome: proportion of participants with Patient Health Questionnaire-9 (PHQ-9) scores of ≥10 in the efficacy analysis set population at baseline, week 7 (postintervention), and month 3 (follow-up). Error bars indicate 95% CIs. Asterisks represent *P*<.05 versus control. AI-iCBT: artificial intelligence–augmented internet-based cognitive behavioral therapy; iCBT: internet-based cognitive behavioral therapy.

Mean PHQ-9 scores decreased significantly from baseline to week 7 in all 3 groups. At week 7, the iCBT group showed a statistically significant improvement compared with the control group (Δ=–1.08, 95% CI –1.98 to –0.18; *P*=.02). However, this difference was not maintained at month 3. Full numerical results are provided in Multimedia Appendix 6. By contrast, the proportion of participants scoring ≥10 on the PHQ-9 decreased only in the iCBT group compared with the control at week 7 (Figure 7). At month 3, the AI-iCBT group showed a significantly lower proportion compared with control (Δ –0.15, 95% CI –0.30 to –0.01; *P*=.046), while the iCBT group did not differ significantly. The group × time interaction was significant (*P*=.008), indicating that the pattern of improvement differed between intervention groups (see Multimedia Appendix 7 for full numerical results).

### Sensitivity Analyses

In evaluating factors associated with low adherence, participants who attended <3 sessions were more likely to be male (65.1% vs 48.4%; *P*<.001), older (mean age 44.2, SD 9.44 vs mean age 41.7, SD 9.55 years; *P*=.001), and employed (287/341, 84.2% vs 212/283, 74.9%; *P*=.02), compared with those who attended ≥3 sessions. No significant differences were observed for marital status, educational background, medical history, or mental health history (Multimedia Appendix 8).

As a further sensitivity analysis, when applying a stricter definition of depression severity—PHQ-9 score of ≥10 plus at least 1 core symptom and SDS score of ≥10—62.7% (178/284) of participants with a PHQ-9 score of ≥10 at baseline met this definition (AI-iCBT: 44/70, 62.9%; iCBT: 41/71, 57.7%; control: 93/143, 65%), with no significant group differences (Multimedia Appendix 9).

## Discussion

### Overview

This study has several unique features. First, it directly compared a fully self-administered CR exercise delivered via iCBT, with and without AI-based NLP functionality, under a randomized (partially masked) design. Notably, the addition of AI led to a statistically significant improvement in engagement—an effect, to our knowledge, not previously documented. As AI-based interventions have become increasingly sophisticated and deeply integrated into intervention programs, disentangling the specific contribution of AI has become difficult. In particular, establishing control conditions that differ only in the presence or absence of AI functionality requires substantial resources, and prior studies have therefore often relied on heterogeneous control conditions. By applying a more robust design—feasible in part because the technology was still in a transitional phase—this trial provides new insights into how AI may enhance fully self-administered iCBT and offers a timely perspective for advancing scalable mental health care.

Self-administered interventions are known to be modestly to moderately effective for depression [15,23], but adherence remains a major limitation [15,18,19,21,55,63]. Systematic reviews indicate that approximately one-third to one-half of participants drop out before completing the program [19,64]. In this context, the engagement improvement observed in this study represents a potential step toward overcoming this barrier.

With regard to clinical effectiveness, no additional benefits of NLP feedback were observed in the ITT population. Recent evidence has reported that greater antidepressant effects are associated with lower dropout rates [15,20,65]. In contrast, although no between-group differences in antidepressant effects were found here, the addition of AI feedback was associated with a statistically significant increase in adherence. This suggests a novel engagement-enhancing mechanism, distinct from the traditionally assumed link between larger clinical effects and lower dropout rates.

Exploratory analyses further indicated that the “empathic function” of AI feedback was significantly associated with improved adherence, whereas the advisory function showed no significant effect. Participants who received empathic responses during their first exercise subsequently demonstrated higher adherence. While self-disclosure was not directly measured, the sense of being supported may have facilitated persistence. These findings align with prior evidence that empathic conversational agents and chatbots support therapeutic alliance and sustained engagement [66-69]. Research in behavioral change has likewise shown that AI-mediated feedback can promote sustained self-management [70], supporting the plausibility of these findings. Such effects of human-AI interaction may have been less visible in prior studies using heterogeneous control conditions but became evident here through the structured randomized design.

Regarding antidepressant effects, no significant between-group differences were observed in the ITT population. In the EAS, results—while requiring cautious interpretation—indicated that at week 7 only the iCBT group showed significant improvement in both the mean PHQ-9 score and the proportion of participants with a PHQ-9 score of ≥10 compared with the control group, whereas the AI-iCBT group did not.

This suggests that the AI function may have attenuated or failed to enhance short-term antidepressant effects. However, this short-term benefit in the iCBT group disappeared at long-term follow-up. For the continuous outcome in particular, the short-term difference was –1.1 points on the PHQ-9, below the minimal clinically important difference (approximately 3 points) [35,71], suggesting limited clinical significance.

By contrast, the short-term dichotomous outcome in the iCBT group represented about a 29% reduction in the proportion of PHQ-9 scores of ≥10 cases relative to the control group. This implies that part of the potential benefit may not have been realized when AI was introduced. At long-term follow-up (month 3), however, only the AI-iCBT group showed a significant reduction of about 15% compared with the control group. These findings highlight the absence of the expected short-term effect in AI-iCBT and the unique long-term effect observed only in AI-iCBT.

The fact that AI-iCBT ultimately demonstrated an effect at long-term follow-up is noteworthy. Although exploratory, this suggests a potential contribution of AI-iCBT in reducing the proportion of participants exceeding a clinically significant severity threshold. One possible explanation is that approaches emphasizing empathy as a core therapeutic skill—such as interpersonal psychotherapy or family therapies—often show slower onset but more enduring gains compared with CBT, lasting well beyond the end of treatment [72-77]. It is possible that the empathy-related function of AI contributed in a similar way, although the underlying mechanisms remain unclear.

The EAS, however, was more restrictive than the ITT population. Among ITT participants with a baseline PHQ-9 score of ≥5, only 149/312 (47.8%) in the AI-iCBT group and 134/312 (42.9%) in the iCBT group attended at least 3 sessions. Furthermore, although a PHQ-9 score of ≥10 is widely recognized as a proxy for “major depression equivalent” in research [33,39], concerns have been raised that it may not be sufficient for diagnostic purposes and may contribute to overdiagnosis [56,78,79]. As a sensitivity analysis, therefore, we used a stricter definition requiring a PHQ-9 score of ≥10 plus at least 1 core symptom (depressed mood or anhedonia) [56,57], together with an SDS score of ≥10 as an indicator of functional impairment [49,58-60]. Results confirmed that only about 62.7% (178/284) of participants who met the PHQ-9 score of ≥10 at baseline also met this stricter definition, highlighting the importance of cautious interpretation (Multimedia Appendix 9).

Taken together, regardless of whether it corresponds to major depression, the finding of a significant reduction in the proportion of participants with clinically meaningful depressive states (PHQ-9 ≥10) compared with the control group may have clinical significance, particularly given the fully self-help nature of the program. From a public health perspective, such a difference could also carry implications for the scalability of self-help programs that do not require therapist involvement.

AI communication, including generative AI, continues to advance rapidly. However, the development of appropriate control programs has often been constrained by logistical and cost-related factors, limiting opportunities to rigorously investigate the antidepressant and anxiolytic effects of AI. Beyond psychiatry, maximizing the effectiveness of self-administered interventions while enhancing engagement remains a critical challenge across health care and welfare domains. This study represents a step toward addressing this challenge.

### Limitations

First, most participants were recruited from a research panel with high affinity for digital interventions. Only 10.9% (129/1187) were actual users of mental health services, which is consistent with the national average in Japan, but caution is required in generalizing these findings to broader populations. Second, high dropout rates were observed, with only 37.6% (149/396) of the AI-iCBT group and 33.8% (134/397) of the iCBT group in the ITT population meeting EAS criteria. Additional analyses indicated that low adherence was associated with being male, older, and employed (Multimedia Appendix 8). Consistent with recent studies, time constraints [22], particularly among employed men [21] and older adults [20,21], were confirmed as key barriers to engagement. Third, although the AI-iCBT group consistently showed 5%-10% higher adherence rates than the iCBT group throughout the trial, both groups had already dropped by half to 50% (396/793) at the very first session and continued to decline over time, remaining at only 30%-40%.

Fourth, missing data were substantial, and MMRM and generalized estimating equations were applied to minimize bias. However, these approaches assume data are missing at random. In this study, since attrition occurred according to participant attributes, the possibility of missing not at random cannot be ruled out, and estimates may remain biased. Fifth, a significant group × time interaction was observed in adherence in the AI feedback group, suggesting a potential role of AI in improving engagement. However, this conclusion is based on a single trial and requires replication in different populations and designs, as well as further elucidation of the underlying mechanisms. Sixth, due to technical issues, session-by-session data on depressive symptoms (Overall Anxiety Severity and Impairment Scale) [80] and anxiety (Overall Depression Severity and Impairment Scale) [81] were lost, precluding more detailed analyses. Future studies should implement automatic data saving and backup systems to prevent such loss. Overall antidepressant effects were small. In addition to limitations of the program itself, this may reflect a ceiling effect due to the predominance of participants with mild depression. Several meta-analyses [23,82,83] report that treatment effects may be less pronounced in cases with mild baseline depression compared to moderate or severe cases. Another limitation is related to our stratified randomization. While stratification by age, sex, and baseline PHQ-9 severity increased internal validity by balancing key prognostic factors, it may also restrict the generalizability of our findings to populations with different distributions of these characteristics.

Despite these challenges, this study provides valuable insights into the potential of AI-enhanced self-help digital interventions, particularly in relation to participant behavior dynamics. Importantly, no major adverse events were reported, underscoring the safety of this innovative approach and its potential to significantly advance mental health care practices.

## Appendix

Multimedia Appendix 1 CONSORT-eHEALTH checklist.

Multimedia Appendix 2 Secondary outcome: ratio of participants with Patient Health Questionnaire-9 scores ≥10 (intention-to-treat population).

Multimedia Appendix 3 Secondary outcomes: mean score of Quick Inventory of Depressive Symptomatology-Japanese version, Generalized Anxiety Disorder-7, and Sheehan Disability Scale (intention-to-treat population).

Multimedia Appendix 4 Satisfaction outcome: Client Satisfaction Questionnaire-8 total scores at week 7 (intention-to-treat population, intervention groups only).

Multimedia Appendix 5 Associations between artificial intelligence feedback functions and exercise attendance (artificial intelligence–augmented internet-based cognitive behavioral therapy and internet-based cognitive behavioral therapy participants, weeks 2–6).

Multimedia Appendix 6 Exploratory outcome: mean Patient Health Questionnaire-9 scores (efficacy analysis set population).

Multimedia Appendix 7 Exploratory outcome: ratio of participants with Patient Health Questionnaire-9 scores ≥10 (efficacy analysis set population).

Multimedia Appendix 8 Associated factors of low adherence in the efficacy analysis set.

Multimedia Appendix 9 Baseline prevalence of major depression proxies in the efficacy analysis set population.

## Abbreviations

AI: artificial intelligence
AI-iCBT: artificial intelligence–augmented internet-based cognitive behavioral therapy
CBT: cognitive behavioral therapy
CONSORT: Consolidated Standards of Reporting Trials
CONSORT-EHEALTH: Consolidated Standards of Reporting Trials of Electronic and Mobile Health Applications and Online Telehealth
CR: cognitive restructuring
CSQ-8: Client Satisfaction Questionnaire-8
EAS: efficacy analysis set
GAD-7: Generalized Anxiety Disorder-7
GLMM: generalized linear mixed models
iCBT: internet-based cognitive behavioral therapy
ITT: intention-to-treat
MMRM: mixed-effects model for repeated measures
NLP: natural language processing
OR: odds ratio
PHQ-9: Patient Health Questionnaire-9
QIDS-J: Quick Inventory of Depressive Symptomatology-Japanese version
SDS: Sheehan Disability Scale

## Data Availability

The data used in this study belong to the Research Institute of Economy, Trade and Industry and can be obtained from the institute upon reasonable request.
