# Supplementary material for: Effect of AI-Based Natural Language Feedback on Engagement and Clinical Outcomes in Fully Self-Guided Internet-Based Cognitive Behavioral Therapy for Depression: 3-Arm Randomized Controlled Trial
Source: J Med Internet Res. 2026 Jan 5;28:e76902. doi: 10.2196/76902 (PMC12817041; doi:10.2196/76902)
Supplement: Multimedia Appendix 1 [file jmir_v28i1e76902_app1.pdf]

CONSORT-EHEALTH Checklist

Note: Due to technical trouble with the JMIR submission system, we were unable to complete the online Google Form version of the CONSORT-EHEALTH checklist. Therefore, we provide the completed checklist in Word format as a Multimedia Appendix. Each item includes direct excerpts from the submitted manuscript (76902-1146925-1-RV 2025-09-23RRR.docx) with section and location indicated.

Item No.	Checklist Item	Addressed?	Excerpt (with location in manuscript)
1a	RCT in title, mode of delivery	Yes	Title: "Artificial Intelligence–Assisted Self-Guided Cognitive Behavioral Therapy for Adults With Depressive Symptoms: Randomized Controlled Trial." (Title, line 1)
1b	Structured abstract incl. adherence/use	Yes	Abstract: "Objective: To evaluate whether AI-augmented iCBT improves engagement and clinical outcomes compared with self-guided iCBT and waiting list. Methods: A three-arm randomized controlled trial…" (Abstract)
2a	Scientific background & rationale	Yes	Introduction: "Depression is a leading global cause of disability… AI-based natural language processing (NLP) may enhance adherence by

			providing feedback.” (Introduction, p.2)
2b	Objectives or hypotheses	Yes	Introduction: “This study aimed to directly compare a fully self-administered cognitive restructuring program delivered via iCBT, with and without AI-based NLP functionality.” (Introduction, last paragraph)
3a	Trial design	Yes	Methods – Trial design: “This study was a three-arm randomized controlled trial, with double-blinding between the AI-iCBT and iCBT groups, and a waiting list control group.” (Methods, p.4)
3b	Changes after trial commencement	No	Not reported. Suggest add: “No major bugs, outages, or changes occurred during the trial.”
4a	Eligibility criteria (incl. IT literacy)	Yes	Methods – Participants: “Participants were aged 20–60 years, scored ≥5 on the PHQ-9, and had regular internet access.” (Methods, p.5)

4b	Settings and locations	Yes	Methods – Procedures: “Participants were recruited from an online research panel and all assessments were completed online.” (Methods, p.5)
5	Interventions	Partial	Methods – Intervention: “The AI-augmented iCBT program, developed by NEC Solution Innovators, Ltd., provided two types of automated feedback: empathetic messages and advisory messages.” (Methods, p.6)<br>Note: No mention of version freeze, QA procedures, or demo URL.
6a	Outcomes	Yes	Methods – Outcomes: “The primary outcome was depressive symptoms measured with the PHQ-9… Engagement was defined as attending at least one session per week.” (Methods, p.7)
6b	Changes to outcomes	Yes	Methods – Sensitivity analyses: “As a sensitivity analysis, a stricter definition of depression severity was used (PHQ-9 ≥10

			plus item thresholds and SDS ≥10).” (Methods, p.8)
7a	Sample size determination	Yes	Methods – Sample size: “A priori sample size was calculated assuming an effect size of d=0.10, with 50% attrition considered.” (Methods, p.9)
7b	Interim analyses/stopping	N/A	Not applicable.
8a/8b/9/10	Randomization, allocation concealment, implementation	Yes	Methods – Randomization and masking: “Randomization was conducted by an independent third party using stratification by age, gender, and baseline PHQ-9.” (Methods, p.10)
11a	Blinding	Yes	Methods – Randomization and masking: “Double-blinding was maintained between AI-iCBT and iCBT groups, but not for the waiting list.” (Methods, p.10)
11b	Similarity of interventions	Partial	Methods – Intervention: “Both iCBT programs were identical except for the AI feedback

			functionality.” (Methods, p.6)
12a	Statistical methods	Yes	Methods – Statistical analysis: “Analyses were conducted using mixed-effects models for repeated measures (MMRM) under the ITT principle… Generalized estimating equations (GEE) were applied for binary outcomes.” (Methods, p.11)
12b	Additional/subgroup analyses	Yes	Results – Exploratory analyses: “Exploratory analyses examined whether empathy versus advisory functions predicted engagement.” (Results, p.14)
13a	Participant flow	Yes	Results: “A total of 1,187 participants were randomized (AI-iCBT: 396, iCBT: 397, WL: 394). Flow is shown in Figure 4.” (Results, p.12)
13b	Losses and exclusions	Yes	Results – Figure 4 shows attrition; reasons for exclusion and dropout are listed.” (Results, p.12, Fig.4)
14a	Recruitment dates	Partial	Results: “Recruitment was conducted between October and December 2023.” (Results, p.12).

			Precise calendar dates not provided.
14b	Why trial ended	Yes	Results: "The trial was completed as planned." (Results, p.12)
15	Baseline data	Yes	Results – Table 1: "Baseline demographic and clinical characteristics are summarized in Table 1. The mean age was 43.5 years, 58.8% male." (Results, p.13, Table 1)
16	Numbers analyzed	Yes	Results: "Analyses were conducted on the ITT population. The EAS population was defined as those attending ≥3 sessions." (Results, p.13)
17a/17b	Outcomes and estimation (continuous and binary)	Yes	Results: "No significant between-group differences were found in PHQ-9 scores… Engagement was higher in AI-iCBT, with odds ratios reported in Table 3." (Results, p.14)<br>Binary PHQ-9: "The proportion of participants with PHQ-9 ≥10 decreased over time in all groups (Figure 5)."
18	Ancillary analyses	Yes	Results – Exploratory analyses: "Empathy

			function in Week 1 predicted subsequent weekly participation (OR=9.99, 95% CI 1.25–79.6).” (Results, p.15)
19	Harms/unintended effects	Partial	Results/Limitations: “No serious adverse events occurred. Technical data loss was noted.” (Results, p.16)<br>Note: No explicit mention that no privacy breaches occurred.
20	Limitations	Yes	Discussion – Limitations: “High dropout rates were observed… most participants were recruited from a research panel… missing data were substantial.” (Discussion, p.18)
21	Generalisability	Yes	Discussion – Generalisability: “Only 12% were actual users of mental health services, limiting generalisability to clinical populations.” (Discussion, p.18)
22	Interpretation	Yes	Discussion – Principal findings: “AI feedback significantly improved engagement but did not enhance clinical effectiveness. This

			highlights both the potential and limitations of AI in self-help interventions.” (Discussion, p.19)
X26	Ethics, consent, privacy	Partial	Methods – Ethical considerations: “The study was approved by the Institutional Review Board… All participants provided electronic informed consent.” (Methods, p.20)<br>Note: Data protection/encryption not detailed explicitly.
