# Supplementary material for: Effect of AI-Based Natural Language Feedback on Engagement and Clinical Outcomes in Fully Self-Guided Internet-Based Cognitive Behavioral Therapy for Depression: 3-Arm Randomized Controlled Trial
Source: J Med Internet Res. 2026 Jan 5;28:e76902. doi: 10.2196/76902 (PMC12817041; doi:10.2196/76902)
Supplement: Multimedia Appendix 2 [file jmir_v28i1e76902_app2.docx]

**Multimedia Appendix 1. Secondary outcome: Ratio of participants with PHQ-9 scores ≥10 (ITT population)**

Proportions at baseline, Week 7, and Month 3 were analyzed using MMRM with binary coding (0/1). Values are LS means with 95% CIs, including between-group comparisons.

| **Time point** | **AI-iCBT group (n=396) LS mean (95% CI)** | **iCBT group (n=397) LS mean (95% CI)** | **Control group (n=394) LS mean (95% CI)** | **AI-iCBT vs Control Δ (95% CI), p** | **iCBT vs Control Δ (95% CI), p** |
| --- | --- | --- | --- | --- | --- |
| Baseline | 0.36 (0.31–0.41) | 0.36 (0.31–0.41) | 0.36 (0.32–0.41) | – | – |
| Week 7 | 0.33 (0.28–0.39) | 0.27 (0.22–0.33) | 0.33 (0.28–0.38) | 0.01 (–0.13 to 0.15), p = .739 | –0.05 (–0.19 to 0.09), p = .143 |
| Month 3 | 0.25 (0.21–0.31) | 0.27 (0.22–0.33) | 0.30 (0.25–0.35) | –0.04 (–0.18 to 0.09), p = .198 | –0.03 (–0.17 to 0.11), p = .409 |
