# Supplementary material for: Effect of AI-Based Natural Language Feedback on Engagement and Clinical Outcomes in Fully Self-Guided Internet-Based Cognitive Behavioral Therapy for Depression: 3-Arm Randomized Controlled Trial
Source: J Med Internet Res. 2026 Jan 5;28:e76902. doi: 10.2196/76902 (PMC12817041; doi:10.2196/76902)
Supplement: Multimedia Appendix 3 [file jmir_v28i1e76902_app3.docx]

**Multimedia Appendix 2. Secondary outcomes: mean score of QIDS-J, GAD-7, and SDS (ITT population)**

Secondary outcomes: mean score of QIDS-J, GAD-7, and SDS at baseline, Week 7, and Month 3 (ITT population), analyzed using MMRM. Values are least-squares means (LS means) with 95% confidence intervals (CI).

| **Outcome / Time point** | **AI-iCBT LS mean (95% CI)** | **iCBT LS mean (95% CI)** | **Control LS mean (95% CI)** | **AI-iCBT vs Control Δ (95% CI)** | **p** | **iCBT vs Control Δ (95% CI)** | **p** |
| --- | --- | --- | --- | --- | --- | --- | --- |
| QIDS-J Baseline | 8.80 (8.52–9.09) | 8.76 (8.47–9.04) | 8.80 (8.52–9.09) | – | – | – | – |
| QIDS-J Week 7 | 7.85 (7.49–8.21) | 7.35 (6.97–7.73) | 8.24 (7.94–8.55) | -0.40 (-0.97–0.18) | .179 | -0.85 (-1.44––0.26) | .005 |
| QIDS-J Month 3 | 7.47 (7.12–7.82) | 7.37 (7.00–7.73) | 7.99 (7.68–8.31) | -0.53 (-1.10–0.05) | .073 | -0.58 (-1.17–0.00) | .051 |
| GAD-7 Baseline | 6.10 (5.83–6.37) | 6.09 (5.82–6.36) | 6.16 (5.89–6.43) | – | – | – | – |
| GAD-7 Week 7 | 5.39 (5.05–5.73) | 5.32 (4.97–5.68) | 5.50 (5.21–5.79) | -0.06 (-0.62–0.50) | .845 | -0.11 (-0.68–0.45) | .693 |
| GAD-7 Month 3 | 4.99 (4.66–5.32) | 4.86 (4.51–5.20) | 5.39 (5.09–5.69) | -0.34 (-0.90–0.22) | .230 | -0.46 (-1.03–0.10) | .108 |
| SDS Baseline | 12.80 (12.32–13.27) | 12.92 (12.45–13.40) | 12.93 (12.45–13.41) | – | – | – | – |
| SDS Week 7 | 12.16 (11.56–12.77) | 11.93 (11.30–12.56) | 12.23 (11.72–12.74) | 0.07 (-0.92–1.05) | .896 | -0.29 (-1.29–0.71) | .567 |
| SDS Month 3 | 12.15 (11.56–12.73) | 11.47 (10.86–12.07) | 12.12 (11.60–12.65) | 0.16 (-0.82–1.14) | .751 | -0.65 (-1.64–0.34) | .200 |
