# Supplementary material for: Effect of AI-Based Natural Language Feedback on Engagement and Clinical Outcomes in Fully Self-Guided Internet-Based Cognitive Behavioral Therapy for Depression: 3-Arm Randomized Controlled Trial
Source: J Med Internet Res. 2026 Jan 5;28:e76902. doi: 10.2196/76902 (PMC12817041; doi:10.2196/76902)
Supplement: Multimedia Appendix 4 [file jmir_v28i1e76902_app4.docx]

**Multimedia Appendix 3. Satisfaction outcome: CSQ-8 total scores at Week 7 (ITT population, intervention groups only)**

User satisfaction assessed with the Client Satisfaction Questionnaire-8 (CSQ-8). Least squares (LS) means with 95% confidence intervals (CI) are presented, analyzed using MMRM. Scores range from 8 to 32, with higher scores indicating greater satisfaction.

| **Outcome** | **AI-iCBT LS mean (95% CI)** | **iCBT LS mean (95% CI)** | **AI-iCBT vs iCBT Δ (95% CI)** | **p** |
| --- | --- | --- | --- | --- |
| CSQ-8 total score | 21.11 (20.39–21.84) | 20.68 (19.93–21.44) | 0.40 (-0.60–1.50) | .417 |
