# Supplementary material for: Effect of AI-Based Natural Language Feedback on Engagement and Clinical Outcomes in Fully Self-Guided Internet-Based Cognitive Behavioral Therapy for Depression: 3-Arm Randomized Controlled Trial
Source: J Med Internet Res. 2026 Jan 5;28:e76902. doi: 10.2196/76902 (PMC12817041; doi:10.2196/76902)
Supplement: Multimedia Appendix 5 [file jmir_v28i1e76902_app5.docx]

**Multimedia Appendix 4. Associations between AI feedback functions and exercise attendance (AI-iCBT and iCBT participants, Weeks 2–6)**

Odds ratios from generalized estimating equation logistic models. All listed covariates were included simultaneously in the adjusted models. This table provides the full version of results; a simplified summary is presented in Table 4 in the main text.

| **Variable** | **Reference Category** | **OR** | **95% CI** | **p-value** |
| --- | --- | --- | --- | --- |
| Empathetic feedback | Never activated | 9.990 | (5.798 to 17.211) | <.001 |
| Advisory feedback | Never activated | 2.370 | (0.963 to 5.833) | .061 |
| Week (continuous) | — | 0.852 | (0.826 to 0.878) | <.001 |
| Empathetic feedback × Week | — | 0.955 | (0.891 to 1.024) | .200 |
| Advisory feedback × Week | — | 0.944 | (0.838 to 1.062) | .335 |
| Group | Standard iCBT | 0.436 | (0.307 to 0.618) | <.001 |
| Gender | Female | 0.602 | (0.452 to 0.803) | <.001 |
| Age (continuous) | — | 0.979 | (0.964 to 0.995) | .010 |
| Marital Status – Married | Never married | 1.170 | (0.848 to 1.616) | .339 |
| Marital Status – Divorced |  | 1.028 | (0.554 to 1.906) | .931 |
| Marital Status – Widowed |  | 2.782 | (0.485 to 15.944) | .251 |
| Education – Junior High School | University or Graduate School | 0.769 | (0.150 to 3.935) | .753 |
| Education – High School |  | 0.544 | (0.380 to 0.778) | <.001 |
| Education – Junior college / Technical school |  | 0.627 | (0.435 to 0.905) | .013 |
| Employment Status – Working | Not working | 1.195 | (0.751 to 1.899) | .452 |
| Employment Status – On leave |  | 1.390 | (0.724 to 2.668) | .322 |
| Mental Treatment – Ongoing | No treatment | 0.899 | (0.585 to 1.381) | .628 |
| Mental Treatment – Past treatment |  | 0.804 | (0.541 to 1.195) | .280 |
| Physical Treatment – Outpatient | No treatment | 1.096 | (0.777 to 1.544) | .602 |
| Physical Treatment – Inpatient |  | 1.191 | (0.299 to 4.743) | .804 |
| Depression Severity (PHQ-9 ≥10) | <10 | 1.121 | (0.840 to 1.495) | .438 |
