# Supplementary material for: Effect of AI-Based Natural Language Feedback on Engagement and Clinical Outcomes in Fully Self-Guided Internet-Based Cognitive Behavioral Therapy for Depression: 3-Arm Randomized Controlled Trial
Source: J Med Internet Res. 2026 Jan 5;28:e76902. doi: 10.2196/76902 (PMC12817041; doi:10.2196/76902)
Supplement: Multimedia Appendix 6 [file jmir_v28i1e76902_app6.docx]

**Multimedia Appendix 5. Exploratory outcome: Mean PHQ-9 Scores (EAS Population)**

Mean PHQ-9 scores at baseline, Week 7, and Month 3, analyzed using a mixed model for repeated measures (MMRM). Values are presented as least-squares means (LS means) with 95% confidence intervals (CI), adjusted for age, gender, medical history, and baseline PHQ-9 score.

| **Outcome / Time point** | **AI-iCBT LS mean (95% CI)** | **iCBT LS mean (95% CI)** | **Control LS mean (95% CI)** | **AI-iCBT vs Control Δ (95% CI)** | **p** | **iCBT vs Control Δ (95% CI)** | **p** |
| --- | --- | --- | --- | --- | --- | --- | --- |
| Baseline | 10.43 (9.88–10.97) | 10.42 (9.85–11.00) | 10.39 (10.02–10.76) | – | – | – | – |
| Week 7 | 8.89 (8.31–9.46) | 8.48 (7.88–9.08) | 9.52 (9.12–9.93) | -0.67 (-1.55–0.20) | .130 | -1.08 (-1.98––0.18) | .019 |
| Month 3 | 8.42 (7.85–8.99) | 8.49 (7.89–9.09) | 9.10 (8.69–9.51) | -0.71 (-1.58–0.15) | .107 | -0.64 (-1.54–0.26) | .164 |
