# Supplementary material for: Effect of AI-Based Natural Language Feedback on Engagement and Clinical Outcomes in Fully Self-Guided Internet-Based Cognitive Behavioral Therapy for Depression: 3-Arm Randomized Controlled Trial
Source: J Med Internet Res. 2026 Jan 5;28:e76902. doi: 10.2196/76902 (PMC12817041; doi:10.2196/76902)
Supplement: Multimedia Appendix 7 [file jmir_v28i1e76902_app7.docx]

**Multimedia Appendix 6. Exploratory outcome: Ratio of participants with PHQ-9 scores ≥10 (EAS population).**

Proportions at each assessment point (baseline, Week 7, Month 3) are shown with 95% confidence intervals. Group, time, and group × time interaction effects were evaluated using generalized linear mixed models (GLMMs) with a logit link, from which estimated proportions and their 95% confidence intervals were obtained.

| **Outcome / Time point** | **AI-iCBT LS mean (95% CI)** | **iCBT LS mean (95% CI)** | **Control LS mean (95% CI)** | **AI-iCBT vs Control Δ (95% CI)** | **p** | **iCBT vs Control Δ (95% CI)** | **p** |
| --- | --- | --- | --- | --- | --- | --- | --- |
| Baseline | 0.49 (0.39–0.59) | 0.51 (0.40–0.60) | 0.48 (0.41–0.55) | – | – | – | – |
| Week 7 | 0.41 (0.31–0.52) | 0.21 (0.12–0.32) | 0.40 (0.33–0.48) | 0.01 (–0.14–0.15) | .960 | –0.21 (–0.36––0.07) | .015 |
| Month 3 | 0.20 (0.12–0.29) | 0.29 (0.19–0.40) | 0.34 (0.27–0.42) | –0.15 (–0.30––0.01) | .046 | –0.08 (–0.22–0.07) | .413 |
