# Supplementary material for: Effect of AI-Based Natural Language Feedback on Engagement and Clinical Outcomes in Fully Self-Guided Internet-Based Cognitive Behavioral Therapy for Depression: 3-Arm Randomized Controlled Trial
Source: J Med Internet Res. 2026 Jan 5;28:e76902. doi: 10.2196/76902 (PMC12817041; doi:10.2196/76902)
Supplement: Multimedia Appendix 8 [file jmir_v28i1e76902_app8.docx]

**Multimedia Appendix 7. Associated factors of low adherence in the EAS**

Baseline characteristics of participants who attended ≥3 sessions compared with those who attended <3 sessions. Adherence was defined as attending at least 3 of 6 sessions. Values are presented as n (%) for categorical variables and mean (SD) for continuous variables. p values are based on independent t tests for continuous variables and chi-square tests for categorical variables.

| **Characteristic** | **≥ 3 times (n = 283)** | **< 3 times (n = 341)** | **p value-1 (t test)** | **p value-2 (chi-square test)** |
| --- | --- | --- | --- | --- |
| **Gender - Male** | **137 (48.4%)** | **222 (65.1%)** |  | **p < .001** |
| Gender - Female | 146 (51.6%) | 119 (34.9%) |  |  |
| **Age - Mean (SD)** | **41.7 (9.55)** | **44.2 (9.44)** | **p = .001** |  |
| Age - Median | 42 | 45 |  |  |
| Age - Min, Max | 20, 60 | 20, 60 |  |  |
| Marital status - Married | 150 (53.0%) | 191 (56.0%) |  | p = .417 |
| Marital status - Divorced | 14 (4.9%) | 22 (6.5%) |  |  |
| Marital status - Bereaved | 3 (1.1%) | 1 (0.3%) |  |  |
| Marital status - Single | 116 (41.0%) | 127 (37.2%) |  |  |
| Education - Junior high school | 1 (0.4%) | 2 (0.6%) |  | p = .893 |
| Education - High school | 60 (21.2%) | 78 (22.9%) |  |  |
| Education - Junior college / Technical / Vocational | 55 (19.4%) | 69 (20.2%) |  |  |
| Education - University / Post-graduate | 167 (59.0%) | 192 (56.3%) |  |  |
| **Employment - Working** | **212 (74.9%)** | **287 (84.2%)** |  | **p = .016** |
| Employment - Unemployed (looking) | 29 (10.2%) | 21 (6.2%) |  |  |
| Employment - Unemployed (not looking) | 42 (14.8%) | 33 (9.7%) |  |  |
| Medical history - No relevant | 219 (77.4%) | 264 (77.4%) |  | p = .089 |
| Medical history - Ambulatory | 58 (20.5%) | 76 (22.3%) |  |  |
| Medical history - Hospitalized | 6 (2.1%) | 1 (0.3%) |  |  |
| Mental history - In treatment | 37 (13.1%) | 47 (13.8%) |  | p = .512 |
| Mental history - Treated | 53 (18.7%) | 52 (15.2%) |  |  |
| Mental history - No relevant | 193 (68.2%) | 242 (71.0%) |  |  |
| PHQ-9 ≥10 | 141 (49.8%) | 144 (42.2%) |  | p = .058 |
| PHQ-9 mean (SD) | 10.5 (4.44) | 10.1 (4.83) | p = .357 |  |
| QIDS-J mean (SD) | 10.2 (4.31) | 9.7 (4.79) | p = .196 |  |
| GAD-7 mean (SD) | 7.0 (4.15) | 6.9 (4.74) |  |  |
