# Supplementary material for: Effect of AI-Based Natural Language Feedback on Engagement and Clinical Outcomes in Fully Self-Guided Internet-Based Cognitive Behavioral Therapy for Depression: 3-Arm Randomized Controlled Trial
Source: J Med Internet Res. 2026 Jan 5;28:e76902. doi: 10.2196/76902 (PMC12817041; doi:10.2196/76902)
Supplement: Multimedia Appendix 9 [file jmir_v28i1e76902_app9.docx]

**Multimedia Appendix 8. Baseline prevalence of major depression proxies in the EAS population.**

Proportions of participants meeting increasingly stringent criteria for probable major depression at baseline are shown: (1) PHQ-9 ≥10, (2) PHQ-9 ≥10 plus presence of a core symptom (item 1 or item 2 ≥2), and (3) PHQ-9 ≥10 plus core symptom plus functional impairment (SDS ≥10). No significant group differences were observed (all p > 0.30).

| **Group** | **Baseline n** | **PHQ-9 ≥ 10 n (%)** | **PHQ-9 ≥ 10 and {Q1 ≥ 2 or Q2 ≥ 2} n (%)** | **% for PHQ-9 ≥ 10** | **PHQ-9 ≥ 10 and {Q1 ≥ 2 or Q2 ≥ 2 and SDS ≥ 10} n (%)** | **% for PHQ-9 ≥ 10** |
| --- | --- | --- | --- | --- | --- | --- |
| AI-iCBT | 149 | 70 (47.0%) | 47 (31.5%) | 67.1% | 44 (29.5%) | 62.9% |
| iCBT | 134 | 71 (53.0%) | 45 (33.6%) | 63.4% | 41 (30.6%) | 57.7% |
| Control | 317 | 143 (45.1%) | 98 (30.9%) | 68.5% | 93 (29.3%) | 65.0% |
